# Small molecules targeting RORγt inhibit autoimmune disease by suppressing Th17 cell differentiation

**DOI:** 10.1038/s41419-020-02891-2

**Published:** 2020-08-22

**Authors:** Jun Tan, Huan Liu, Minhao Huang, Na Li, Shibing Tang, Jiayu Meng, Shiyun Tang, Hongxiu Zhou, Aize Kijlstra, Peizeng Yang, Shengping Hou

**Affiliations:** 1 The First Affiliated Hospital, Chongqing Medical University, Chongqing Key Laboratory of Ophthalmology, Chongqing, P. R. China; 2 Guangzhou Institutes of Biomedicine and Health, Chinese Academy of Sciences, Guangzhou, 510530 China; 3 College of Basic Medicine, Chongqing Medical University, Chongqing, 400016 P. R. China; 4 Eye Research Institute Maastricht, Department of Ophthalmology, University Hospital Maastricht, Maastricht, The Netherlands

**Keywords:** Virtual screening, Autoimmunity

## Abstract

Th17 cells, a lymphocyte subpopulation that is characterized by the expression of the transcription factor “retinoic acid receptor-related orphan receptor gamma-t” (RORγt), plays an important role in the pathogenesis of autoimmune disease. The current study was set up to discover novel and non-steroidal small-molecule inverse agonists of RORγt and to determine their effects on autoimmune disease. Structure-based virtual screening (SBVS) was used to find compounds targeting RORγt. Flow cytometry was used to detect the Th17 cell differentiation. Inverse agonists were intraperitoneally administered to mice undergoing experimental autoimmune uveitis (EAU), experimental autoimmune encephalomyelitis (EAE) or type 1 diabetes. The effects of the inverse agonists were evaluated by clinical or histopathological scoring. Among 1.3 million compounds screened, CQMU151 and CQMU152 were found to inhibit Th17 cell differentiation without affecting the differentiation of Th1 and Treg lineages (both *P* = 0.001). These compounds also reduced the severity of EAU (*P* = 0.01 and 0.013) and functional studies showed that they reduced the number of Th17 cell and the expression of IL-17(Th17), but not IFN-γ(Th1) and TGF-β(Treg) in mouse retinas. Further studies showed that these compounds may reduce the expression of p-STAT3 by reducing the positive feedback loop of IL-17/IL-6/STAT3. These compounds also reduced the impaired blood–retinal barrier function by upregulating the expression of tight junction proteins. These compounds were also found to reduce the severity of EAE and type 1 diabetes. Our results showed that RORγt inverse agonists may inhibit the development of autoimmune diseases and may provide new clues for the treatment of Th17-mediated immune diseases.

## Introduction

Th17 cells, as a third subset of Th cells, are effective tissue inflammation inducers and are involved in the pathogenesis of many inflammatory and autoimmune diseases^1^. Th17 cells not only play an important role in host defense against bacterial and fungal infections but also in many immune-related diseases, including uveitis, psoriasis, rheumatoid arthritis, multiple sclerosis, inflammatory bowel disease, and asthma/airway inflammatory disease^2,3^. Th17 cells mediate inflammation by producing IL-17^2^. Anti-IL-17 has been shown to have a good therapeutic effect on a variety of human diseases^4,5^. In Th17 cells, IL-17 transcription is regulated by the Th17-specific transcription regulator, RORγt^6–8^. RORγt is a ligand-dependent transcriptional factor and belongs to the nuclear hormone receptor superfamily^9^. The discovery of non-steroidal small-molecule RORγt inverse agonists has become one of the intriguing research areas for treating Th17-mediated autoimmune diseases. Two natural products including Digoxin and Ursolic acid were both shown to suppress IL-17 production by selectively inhibiting RORγt^10,11^. However, digoxin has carcinogenic and toxic side effects and ursolic acid also work on other targets, including the glucocorticoid receptor^12,13^. Thus, further studies are needed to find more specific RORγt inverse agonists. A series of inverse agonists including SR1001, SR2211, and SR1555 were then developed based on a similar scaffold^14–16^. SR1001 treatment was found to delay the onset and clinical severity of EAE and type 1 diabetes^14,17^. SR2211 could have a potential utility for the treatment of collagen-induced arthritis (CIA)^18^. In addition, TMP778, a RORγt inverse agonist, selectively regulated Th17-signature gene expression in mononuclear cells and then reduced imiquimod-induced psoriasis-like cutaneous inflammation as well as EAU^19,20^. These studies imply that RORγt inverse agonist could be considered as a new attractive strategy for autoimmune disease. Herein, we performed structure-based virtual screening targeting the RORγt ligand-binding domain. Nearly 1.3 million compounds were screened and only two compounds, Z26782034 (CQMU151) and Z167626774 (CQMU152) were shown to effectively inhibit Th17 cell differentiation and were able to reduce the severity of experimental autoimmune disease models such as EAU, EAE, and type 1 diabetes.

## Methods

### Animals

Female C57BL/6J mice, at 6–8 weeks of age, were provided by the Experimental Animal Center of Chongqing Medical University and were housed in a specific pathogen-free facility. All procedures were carried out conforming to the ARVO Statement for the Use of Animals in Ophthalmic and Vision Research. The protocol was approved by the Ethics Committee of the First Affiliated Hospital of Chongqing Medical University. The sample size is estimated based on the sum of the minimum sample sizes required for each experiment. All experimental animals are grouped by simple random sampling method. The scores of animal models are evaluated blindly.

### Reagents

Human IRBP_651-670_(LAQGAYRTAVDLESLASQLT) (purity 98%) was synthesized by Shanghai Sanyuan Bioengineering Technology and Services Co., Ltd. (Shanghai, China). Complete Freund’s adjuvant was purchased from Sigma-Aldrich (St. Louis, Missouri, USA). Heat-killed M. tuberculosis strain H37Ra was purchased from BD Biosciences (New Jersey, USA) and pertussis toxin (PTX) was purchased from Sigma-Aldrich (St. Louis, Missouri, USA). Small-molecule compounds were purchased from Enamine (Ukraine).

### Virtual screening

The crystal structure of RORγt protein’s ligand-binding domain in complex with an antagonist was taken from the Protein Data Bank (PDB code: 4QM0) (The structures of LBD are the same between RORγt and RORγ) and was used for molecular docking. Protein structure optimization including hydrogen atoms addition, water deletion, protonation state adjustment, and restrained minimization were processed using the “Protein Preparation Wizard” workflow in Maestro (version 9.4, Schrödinger, LLC, New York, NY, 2013) with default parameters. The 1.3 million compounds of ChemBridge database were selected and downloaded from the ChemBridge website (https://www.chembridge.com/screening_libraries/) for virtual screening. The 3D structure of inhibitors was prepared using the LigPrep module of Maestro with OPLS3e force field. Glide module was used as the docking program using HTVS, Glide SP and Glide XP modes. Glide HTVS docking output the top 0.1 million structures for further Glide SP docking, and then the top 10000 poses were docked with XP mode. The top-ranked 200 compounds with XP GScore lower than −9 were clustered for visual inspection, and 15 compounds were selected and purchased for further biological evaluation.

### EAU induction and treatment

Female C57BL/6J mice were subcutaneously immunized with 500 μg of human IRBP651–670 dissolved in PBS, emulsified with an equal volume of complete Freund’s adjuvant containing M. tuberculosis strain H37Ra (1:1, v/v). At the same time, the mice received 1 μg of B. pertussis toxin intraperitoneally. Because some mice might fail to develop EAU, we immunized 18 mice per group to ensure that we would have 16 mice for further experiments. Mice were randomly assigned to the naive untreated group, the vehicle group (placebo injection), and compounds intervention groups. The small-molecule compounds were diluted with water containing 30% PEG 300 (Sigma-Aldrich, St. Louis, Missouri, USA) and 1% Tween-80 (Sigma-Aldrich, St. Louis, Missouri, USA). Mice were treated daily with an intraperitoneal injection (Mice in the vehicle group were injected with solvent only, and mice in CQMU151 group or CQMU152 group were injected with CQMU151 or CQMU152 dilution, respectively.) at a dose of 25 mg/kg for 5 days starting from the 9th day after immunization. Prior to inclusion in the experiments all immunized mice were inspected by slit lamp to ensure they had developed uveitis. The dose of 25 mg/kg was based on a similar dose regimen used in other studies in this field^14,21,22^. The control groups were injected with the solvent in the same manner. A separate control group included mice that had been injected with the adjuvant without IRBP. On day 14 and 21 after immunization, the clinical symptoms of EAU were examined by a slit lamp and fundus imaging. The clinical severity of ocular inflammation was assessed in a masked manner by two independent observers and scored on a 0.5-point scale (in half-point increments) based on five independent criteria and Caspi’s criteria^23,24^. During the experiment, we did not observe any unnatural animal deaths.

### Luciferase reporter gene assay

The CNS2-Il17a and RORE reporter constructs were transfected to 293T cells for dual-luciferase reporter gene assays^6^. Reporter gene activity was normalized to Renilla luciferase activity.

### Induction of Th17 cells in vitro

Spleen and lymph nodes were taken from C57BL/6J mice, and naive CD4^+^ T cells were isolated by magnetic sorting using naive CD4^+^ T Cell Isolation Kit (Miltenyi Biotec, Germany). Cells were seeded in a 24-well plate at 0.5 × 10^6^/well (coated with 2 μg/ml anti-mouse CD3 (Biogems, CA, USA) and anti-mouse CD28 (Biogems) the day before to stimulate cell proliferation). Th17 cell differentiation was induced by adding 20 ng/ml IL-1β (Peprotech, NJ, USA), 20 ng/ml IL-23(Biolegend, San Diego, CA, USA), 25 ng/ml IL-6 (Peprotech), and 3 ng/ml TGF-β (Peprotech)^25,26^. At the same time, small-molecule compounds were added at a final concentration of 0, 10, 50, 100 μM. Three days later, the cells were collected to determine the frequency of Th17 cells by flow cytometry. An improved method during rescreening: The Th17 cell inducing factors were added again with the same concentration the next day after seeding to induce more Th17 cells, so that the inhibitory effect of the compound is more prominent.

### Flow cytometry analysis

Mouse spleens were removed, crushed in a cell strainer (40-μm nylon, BD Falcon, San Jose, CA, USA) and filtered to obtain a single-cell suspension. For intracellular staining, cells were stimulated with a cell activation cocktail (with Brefeldin A) (Biolegend, San Diego, CA, USA) for 5 h. Then cells were collected and stained with anti-mouse CD4 FITC (Biolegend), anti-mouse IL-17 PE (Biolegend), anti-mouse IFN-γ PerCP-Cy5.5 (Biolegend), and anti-mouse FOXP3 Alexa Fluor 647 (Biolegend) antibodies. Cells were then analyzed with BD FACSAria (USA), and the acquired data were analyzed with FlowJo X.

### Hematoxylin and eosin (H&E) staining

Sixteen mice each group were euthanized on days 14 or 21 after immunization, and then the eyeballs were enucleated, fixed and immersed in PBS with 10% formaldehyde and 5% glacial acetic acid. The dehydrated tissue was embedded in paraffin wax and sectioned to 4–6-μM thickness. Sections were stained with H&E, and each eye was scored according to Caspi’s criteria^24^.

### Western blotting

Western blotting was performed using standard methods. The retina was isolated from the eye. Protein was extracted using the nuclear and cytoplasmic protein extraction kit (Beyotime, Shanghai, China), and the protein concentration was measured using a bicinchoninic acid assay kit (Beyotime, Shanghai, China). The protein concentration was measured using a bicinchoninic acid assay kit, and an equal amount of 40 μg protein was separated by 6–15% SDS-polyacrylamide gel electrophoresis. The separated proteins were electrotransferred onto polyvinylidene difluoride membranes (Millipore, MA, USA). The membranes were blocked with 5% skim milk, and incubated with primary antibodies for 16 h at 4 °C. Primary antibodies used included anti-IL-17 (1:500, Abcam, UK), anti-IFN-γ (1:500, Bioss, Beijing, China), anti-TGFβ (1:500, Abcam), anti-claudin-5 (1:1000, Abcam), anti-ZO-1 (1:250, Proteintech, Wuhan, China), anti-phospho-STAT3 (1:500, Abcam), anti-STAT3 (1:500, Abcam), and anti-SOCS3 (1:400, Abcam). GAPDH (1:10,000, Proteintech) was used as an internal reference. The membrane was washed with Tris-buffered saline with 0.1% Tween-20 and incubated with HRP-affinipure goat anti-rabbit IgG or HRP-affinipure goat anti-mouse IgG secondary antibodies (1:5000, Proteintech) for 1 h at room temperature. The membrane was visualized by using an ECL kit (Advansta, CA, USA) and band densitometry was quantified using Image J software. Because the molecular size of some target genes is close, so after the visualization of IL-17 and SOCS3, we washed the membrane with antibody clearance solution (Beyotime, Shanghai, China). Then menbrane was blocked and incubated the IFN-γ and claudin-5 primary antibodies.

### BRB integrity using Evans blue leakage

Hundred microliters of 2% (w/v) Evans Blue dye (Sigma-Aldrich, St. Louis, Missouri, USA) was injected through the tail vein. After 2 h, the mice were euthanized; the eyeballs were removed, and immersed in 4% paraformaldehyde for 2 h. The cornea, lens, sclera, and vitreous were removed and then the retina was carefully stripped and washed in cold PBS. Finally, the retina was placed as a flatmount on the slide with the vitreous facing up and mounted with glycerol. Photographs were taken using an immunofluorescent microscope (Leica, Germany). The fluorescence intensity was quantified using Image J software (Germany).

### Immunofluorescence

Eyes were removed on day 14, immediately immersed in 4% paraformaldehyde for 2 h, and then the retina was placed on the slide as a flatmount according to the method described above for Evans blue, and blocked with a PBS solution containing 5% goat serum and 1% Triton-X for 2 h. The retina was incubated with isolectin B4 FITC conjugate (Sigma-Aldrich, St. Louis, Missouri, USA), CD4 (1:500, Elabscience, Wuhan, China) and IL-17 (1:200, Abcam, UK) at 4 °C overnight. After washing, it was incubated with DyLight 405-conjugated affinipure Goat anti-Rabbit IgG (H+L) (1:2500, Rockland, PA, USA) and PE-conjugated Goat anti-mouse IgG (H+L) (1:1000, Multi Science, Hangzhou, China) for 2 h at room temperature. Photos were taken with a confocal microscope (Zeiss, Germany). CD4 and IL-17 dual stained cells were marked as Th17 cells. We took 3–5 high magnification fields for each retina to count Th17 cells (only counted the cells infiltrating into the retina), and measured the average value for statistical analysis.

### Induction and evaluation of the EAE model

According to the pre-experimental modeling success rate and disease severity, ensure that 6 or more mice are successfully induced in each group. Mice were injected subcutaneously with 200 μg of MOG35-55 peptide emulsified in complete Freund’s adjuvant to induce EAE. At the same time, 200 ng of PTX was injected intraperitoneally and re-injected the next day after immunization. The disease score ranged from 0 to 5; 0, none; 1, a weak tail or toddler; 2, gait swing; 3, hind limb paralysis; 4, hind limb and forelimb paralysis; 5, death. When symptoms appeared on the 13th day, successfully modeled mice were randomly assigned to each group and injected intraperitoneally with 25 mg/kg inhibitor or solvent for 5 consecutive days. Spinal cord sections were collected on the 18th day, with a pathological score ranging from 0 to 4; 0, no inflammatory infiltration; 1, inflammatory cell infiltration is limited to the blood vessels and the spinal cord; 2, mild inflammatory cell infiltration in the spinal parenchyma; 3, moderate infiltration; 4, severe infiltration. When scoring, the pictures of all mice were arranged in disorder by someone other than the rater. The rater did not know the group to which the mouse was rated.

### Induction and evaluation of the type 1 diabetes model

Same as the other two models, according to the pre-experimental modeling success rate, 6 mice were successfully induced in each group. After the mice were fasted for 12 h, STZ (Sigma-Aldrich, St. Louis, Missouri, USA) was dissolved in a 0.1 M buffer solution prepared by mixing citric acid and trisodium citrate to form a 10 mg/ml solution. After filtering, the mice were injected intraperitoneally at a dose of 200 mg/kg. Two hours later, glucose water was administered by gavage at 1 g/kg. From the third day, the body weight, food intake and water consumption of the mice were recorded daily. Blood glucose was measured at day 7.

### Statistical analysis

All data are presented as the mean ± s.d. and were analyzed by SPSS 20.0 software (IBM, Chicago, IL, USA). The unpaired Student’s *t*-test was applied to assess significance between two groups. Experimental data for multiple group comparisons were analyzed by one-way ANOVA. Bonferroni post hoc analysis was used after one-way ANOVA. The Kruskal–Wallis test was used for multiple group comparisons that did not comply with normal homogeneity and homogeneity of variance. The differences were considered to be statistically significant at *P* < 0.05.

## Results

### Structure-based virtual screening of small molecules which target RORγt protein

**Fig. 1:**
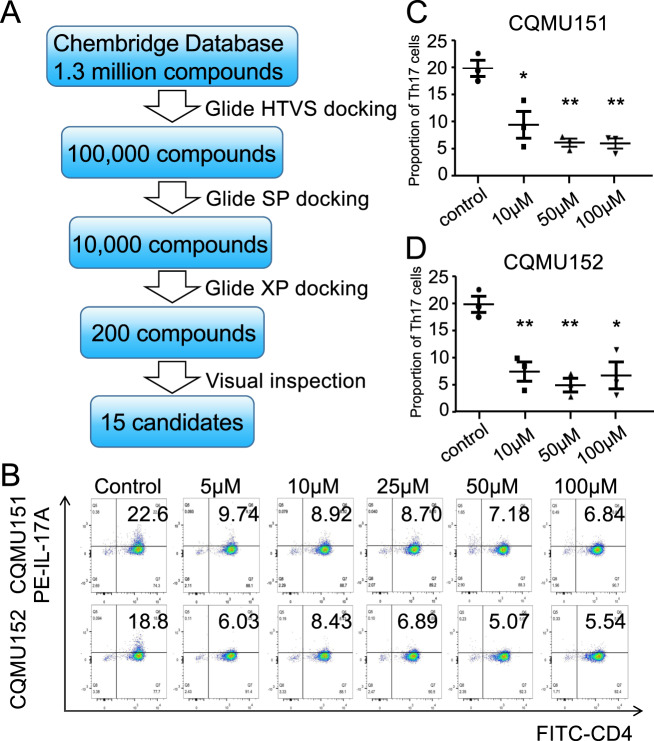
Virtual screening process and in vitro screening. **a** Flowchart of structure-based virtual screening for RORγt inverse agonist discovery. **b** Representative graphs of multiple screening results of CQMU151 and CQMU152 at a concentration of 5, 10, 25, 50, and 100 μM. **c**, **d** Statistics of three repeated experiments of CQMU151 and CQMU152 at concentrations of 10, 50, and 100 μM (*P < 0.05, **P < 0.01 versus the control group with Student’s *t*-test, *n* = 3).

Molecular docking in the high-throughput virtual screening (HTVS) mode was performed on nearly 1.3 million compounds from the ChemBridge Corp. database. Compounds from the top 10% of the ranking list were selected for two further rounds of docking calculations (Fig. 1a). Among these candidates, 15 compounds were selected based on their XP GScore and binding mode analysis (Supplementary Table 1) and were purchased for further biological function evaluation.

### Small molecules obtained by screening can inhibit Th17 differentiation

**Fig. 2:**
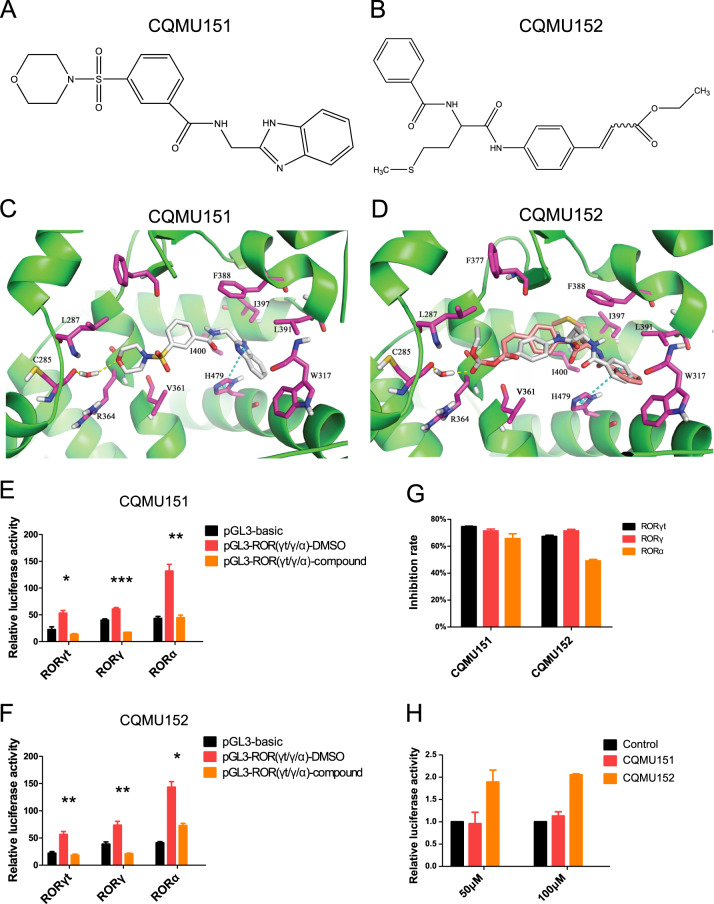
Information about the selected compounds and the target verification. **a**, **b** Structural formulas of CQMU151 and CQMU152, respectively. **c**, **d** The predicted binding modes of CQMU151 and CQMU152 in the RORγt protein’s ligand-binding domain. Key residues are shown as purple sticks. Hydrogen bond interaction between compounds and protein is represented as a yellow dashed line. π–π interactions are represented as cyan dashed lines. CQMU151 is displayed in gray sticks. *Cis*- (9Z) and *trans*- (9E) isomer of CQMU152 are displayed in pink and gray sticks, respectively. **e**, **f** Results of dual-luciferase report assays of CQMU151 and CQMU152, respectively. pGL3-basic represents the negative control of untransfected IL-17 promoter and transcription factor overexpression plasmid, pGL3-RORγt/γ/α-DMSO represents the co-transfection of the promoter plasmid and three overexpression plasmids of RORγt, RORγ, and RORα, respectively, and then added an equal volume of DMSO to the compound as vehicle. pGL3-γt/γ/α-compound represents that compound intervention was added after co-transfection (*P < 0.05, **P < 0.01, ***P < 0.001 versus the DMSO group with Student’s *t*-test). **g** Inhibition rate of two compounds on RORγt, RORγ, and RORα (***P < 0.001 versus the RORα group with one-way ANOVA, post hoc analysis using Bonferroni, *n* = 3). **h** Test of the toxicity of two compounds on naive CD4^+^T cells by CCK8. It can be seen that neither compound caused a significant reduction in the number of cells.

Naive CD4^+^ T cells were isolated from mouse spleen (identification purity above 95%, data not shown) and cultured with IL-1β, IL-6, IL-23, and TGF-β to induce Th17 differentiation^27^. Fifteen candidates from the SBVS were added to the cultures and flow cytometry showed that 5 of them inhibited the induction of Th17 differentiation with an inhibition rate of ~50% (Supplementary Table 2). We then performed repeated experiments on these five compounds using a modified method (stimulate again at the next day to induce more Th17 cells). The results showed that two small-molecule compounds CQMU151 (Z26782034) (EC_50_ = 13.43 μM) and CQMU152 (Z167626774) (EC_50_ = 22.21 μM) showed a consistent inhibition of Th17 differentiation (Fig. 1b–d). Here we showed the molecular formulas of the two compounds (Fig. 2a, b). Molecular docking predicted the binding modes of CQMU151 and CQMU152 in the Ligand-binding domain (LBD) of RORγt (Fig. 2c, d). It shows that both compounds bind to the LBD of RORγt tightly in a similar binding mode. Both the oxygen atom of the morpholine moiety of CQMU151 and the oxygen atom of the ethyl acetate moiety of the *trans-*isomer of CQMU152 form a hydrogen bond interaction with the Cys285 of the RORγ protein via a water molecule. π–π interactions between compounds and His479 of RORγ protein were also seen in the predicted binding modes. The hydrophobic interactions between the benzimidazole moiety of CQMU151 and the phenyl moiety of CQMU152 with the hydrophobic cavity of RORγt formed by Ile397, Leu391, W317, and His479 also stabilize binding of molecules with RORγt protein. Similar Hydrogen bond interaction and π-π interactions were found in other selected candidate compounds (Supplementary Fig. 1).

In order to verify the Th17 specificity of the compounds, we also tested the two compounds on their ability to affect Th1 and Treg cell differentiation. No effect on Th1 and Treg cell lineages could be detected (Supplementary Fig. 2). We also performed a dual-luciferase report assay to verify the target of the compounds. The results showed that both compounds could inhibit the activity of RORγt and retinoic acid receptor-related orphan receptor gamma (RORγ), because RORγ has the same LBD structure as RORγt. Unexpectedly, they also inhibited retinoic acid receptor-related orphan receptor alpha (RORα) (Fig. 2e, f). The inhibitory rate of the compounds on three transcription factors is shown in Fig. 2g. A CCK8 toxicity test showed that the inhibitory effect on Th17 differentiation was not due to possible cytotoxic effects of these compounds (Fig. 2h).

### CQMU151 and CQMU152 inhibits the development of EAU

**Fig. 3:**
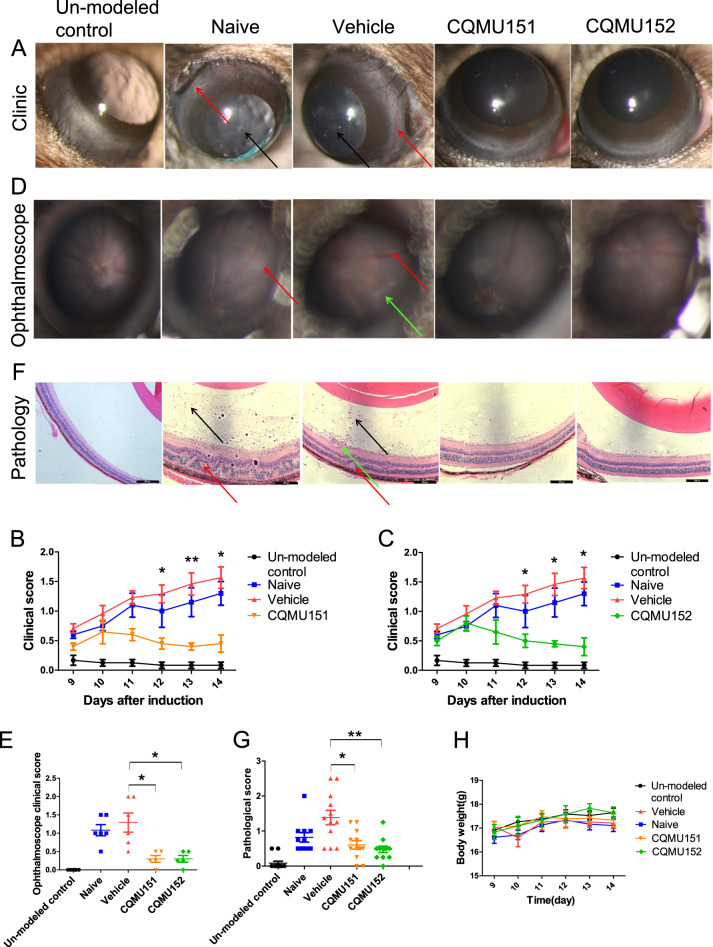
CQMU151 and CQMU152 reduce inflammation of EAU. **a** Representative picture of slit lamp photography on day 14 of the mice in each group. Black arrows represent anterior chamber inflammatory cell infiltration, and red arrows represent conjunctival and/or ciliary congestion. **b**, **c** Clinical score statistics of CQMU151 and CQMU152, respectively (*P < 0.05, **P < 0.01, versus the vehicle group with one-way ANOVA (day 12) or Kruskal–Wallis (days 13 and 14), vehicle group and un-modeled control: *n* = 6, naive, CQMU151 and CQMU152: *n* = 5). **d** Representative picture of fundus photography on day 14 of the mice in each group. Red arrows represent small focal lesions and black arrows represent linear lesion. **e** Fundus photography score statistics of CQMU151 and CQMU152 (*P < 0.05, versus the vehicle group with one-way ANOVA, post hoc analysis using Bonferroni. Naive group, vehicle group and un-modeled control: *n* = 6, CQMU151 and CQMU152: *n* = 5). **f** H&E staining of eyeball sections of the mice in each group on day 14. Black arrows represent vitreous inflammatory cell infiltration, red arrows represent retinal folds and other structural damage, and green arrows represent vasculitis. Scale bar, 203 µm. **g** The graph is the pathological score statistics (*P < 0.05, **P < 0.01 versus the vehicle group with Kruskal–Wallis, naive group: *n* = 11, vehicle, un-modeled control, CQMU151 and CQMU152: *n* = 12). **h** Weight change of the mice of each group. The weight of each mouse was recorded daily from day 9 to 14.

Because Th17 cells are the causative lymphocyte population for the development of EAU, we tested whether CQMU151 and CQMU152 could affect this experimental model of intraocular inflammation. C57BL/6J mice were immunized with interphotoreceptor retinoid binding protein (IRBP) to induce EAU. On the 9th day after IRBP immunization, the clinical symptoms of EAU were examined with a slit lamp. Mice with ciliary hemorrhage and/or inflammatory cell infiltration in the anterior chamber were selected for treatment with the two selected compounds. A slit lamp was used to observe the anterior chamber every day from the 9th day after immunization. From this time point on we started a 5-day period of continuous treatment. The mice were scored by clinical and histopathological examination on day 14 after immunization. After CQMU151 and CQMU152 treatment, the conjunctival and ciliary hyperemia as well as infiltration of anterior chamber inflammatory cells were significantly decreased. The slit lamp score was also significantly reduced (Fig. 3a–c). Fundus photography results showed that after compounds treatment, the retinal vasculitis was significantly lower, and the fundus score was significantly reduced (Fig. 3d, e). Histopathological sections showed that the treated mice had less retinal folds and less inflammatory cells as compared with the control groups (Fig. 3f, g). Our results also showed that CQMU151 and CQMU152 decreased EAU scores but did not delay the onset of EAU (Supplementary Fig. 3). In addition, the mice were well tolerated to the treatment of the two compounds without obvious body weight loss (Fig. 3h).

### CQMU151 and CQMU152 regulates the proportion of Th17 cells in vivo

**Fig. 4:**
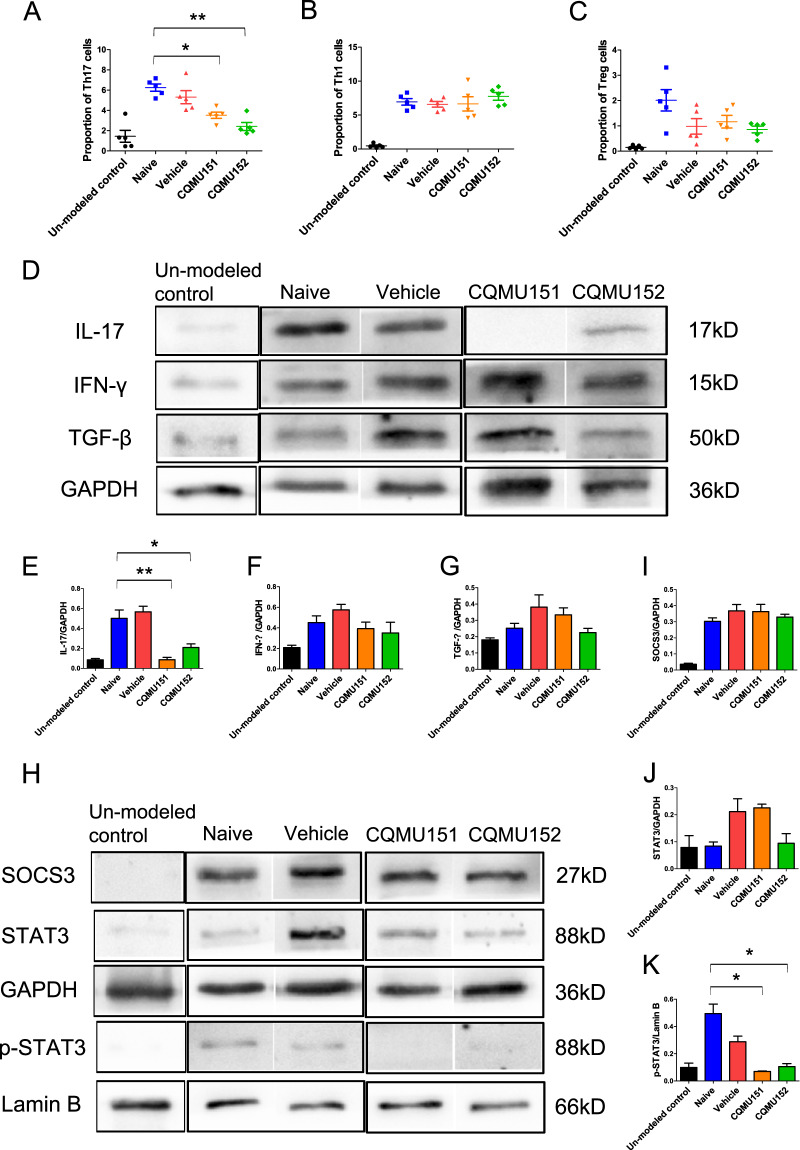
CQMU151 and CQMU152 worked by reducing IL-17 expression. **a**–**c** Flow cytometry results of Th17 cells, Th1 cells, and Treg cells in the spleen and lymph node on the 14th day of each group (*P < 0.05, **P < 0.01 versus the vehicle group with one-way ANOVA, post hoc analysis using Bonferroni, *n* = 5). **d** Representative western blots of IL-17, IFN-γ, and TGF-β in the mouse retina of mice from each group on day 14. **e**–**g** Quantification of the relative fold change of IL-17, IFN-γ, and TGF-β (*P < 0.05, **P < 0.01 versus the naive group with one-way ANOVA, post hoc analysis using Bonferroni*, n* = 3). **h** Representative western blots of retinal SOCS3, STAT3, and p-STAT3 of mice from each group on day 14. **i**–**k** Quantification of the relative fold change of SOCS3, STAT3, and p-STAT3 (*P < 0.05, versus the naive group with one-way ANOVA, post hoc analysis using Bonferroni, *n* = 3. In p-STAT3 statistics: naive, vehicle group: *n* = 4, un-modeled control and compounds groups: *n* = 3).

Because the two compounds tested target Th17 transcription factors, we performed flow cytometry on mouse spleen T cell subsets at day 14 following immunization with IRBP. The frequency of splenic CD4^+^IL-17^+^Th17 cells in mice treated with these compounds was significantly decreased as compared to the controls (Fig. 4a). However, the frequency of CD4^+^IFN-γ^+^Th1 cells and CD4^+^FOXP3^+^Treg cells were not significantly different from controls (Fig. 3b, c). To verify this result, we also collected mouse retinas on day 14 after immunization to measure protein expression levels of IL-17, IFN-γ, and TGF-β. The results showed consistent results with the flow cytometry analysis of splenic cells. Only IL-17 protein expression was decreased in the two treatment groups (Fig. 4d–g).

### CQMU151 and CQMU152 may affect the IL-17/IL-6/p-STAT3 feedback loop

Signal transducer and activator of transcription (STAT) and suppressor of cytokine signaling (SOCS) play important roles in cell signal transduction and the differentiation of Th17 cells^28^. We tested the retinal protein expression of SOCS3, STAT3, and p-STAT3 and showed that p-STAT3 was significantly lower in the treatment groups compared to controls. Treatment however did not affect SOCS3 expression (Fig. 4h–k).

To investigate the possibility that CQMU151 and CQMU152 directly inhibit STAT3 and to study possible off-target effects, molecular docking studies of COMU151 and CQMU152 against the STAT3 SH2 domain were investigated, which would block Y705 phosphorylation and further prevent dimerization and transcriptional activity of STAT3^29,30^. The results showed that the ligand-binding pocket of STAT3 is totally different from that of RORγt. Since the ligand-binding pocket of the STAT3 SH2 domain is located in the surface of the protein, more interaction between compounds and amino acids of the protein are needed to stabilize their binding. The molecular docking showed a weak binding between the STAT3 SH2 domain and both CQMU151 and CQMU152 through two and one hydrogen bond interactions, respectively, which could not result in a sustained binding of compounds against STAT3 like other STATS inhibitors do (Supplementary Fig. 4). GScore analysis of CQMU151 and CQMU152 against STAT3 also revealed poor binding between both compounds and STAT3 (Supplementary Table 3). Based on the above evidence, we suggest that it is unlikely that the compound directly inhibits STAT3.

### CQMU151 and CQMU152 can prevent the breakdown of the BRB in EAU and reduce IL-17^+^ lymphocyte infiltration

**Fig. 5:**
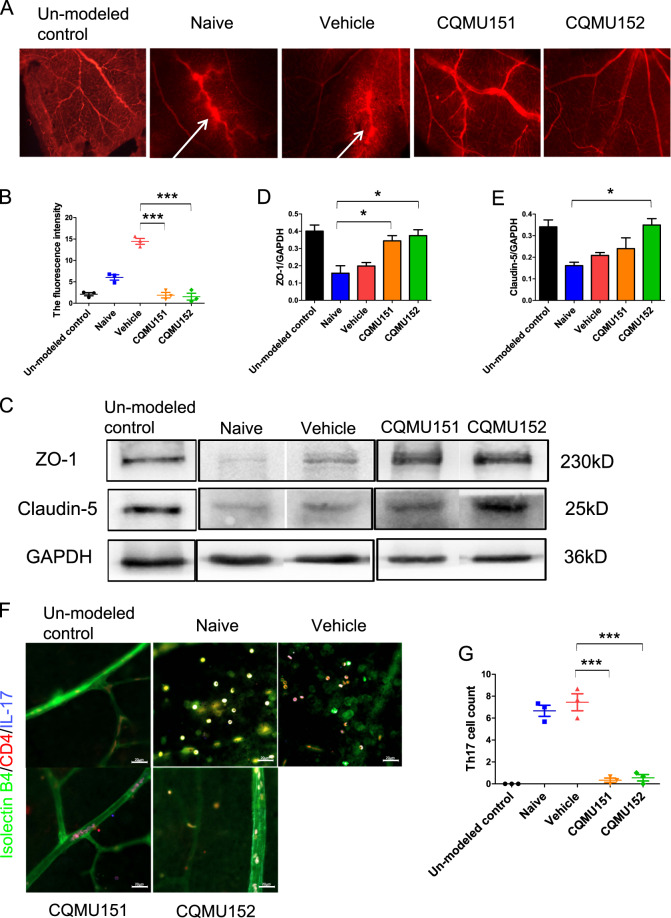
CQMU151 and CQMU152 maintain BRB integrity. **a** Retinal flat-mount images of retinal Evans blue leakage. Scale bar, 80 µm. **b** Quantitative statistics of fluorescence intensity of (**a**) (***P < 0.001, versus the vehicle group with one-way ANOVA, post hoc analysis using Bonferroni, *n* = 3). **c** Representative Western blots of ZO-1 and Claudin-5 in the retina of the mice from each group on day 14. **d**, **e** Quantification of the relative fold change of ZO-1 and Claudin-5 (*P < 0.05, versus the naive group with one-way ANOVA, post hoc analysis using Bonferroni, *n* = 3). **f** Immunofluorescence co-staining of retinal flat mounts with isolectin B4 (green), IL-17 (blue), and CD4 (red). Scale bar, 50 µm. Arrows indicate Th17 cells infiltrated into the retina co-stained by CD4 and IL-17. **g** Statistics of Th17 cells infiltrating into the retina (***P < 0.0001, versus the vehicle group with one-way ANOVA, post hoc analysis using Bonferroni, *n* = 3).

To examine the effect of compounds CQMU151 and CQMU152 on the breakdown of the blood–retinal barrier (BRB), we measured the integrity of BRB using Evans blue leakage from retinal blood vessels. Diffuse leakage of the dye was significantly relieved in the treatment groups (Fig. 5a, b). Similar to the aforementioned results, the expression of a tight junction protein in the retina was increased in the treated groups compared to the control group (Fig. 5c–e), suggesting that the two compounds may protect or repair BRB breakdown. Immunofluorescence microscopy showed a marked decrease in the amount of infiltrating retinal Th17 cells in the treated groups, suggesting that BRB breakdown is associated with the presence of these cells (Fig. 5f, g).

### CQMU151 and CQMU152 inhibit the development of Th17-mediated diseases

**Fig. 6:**
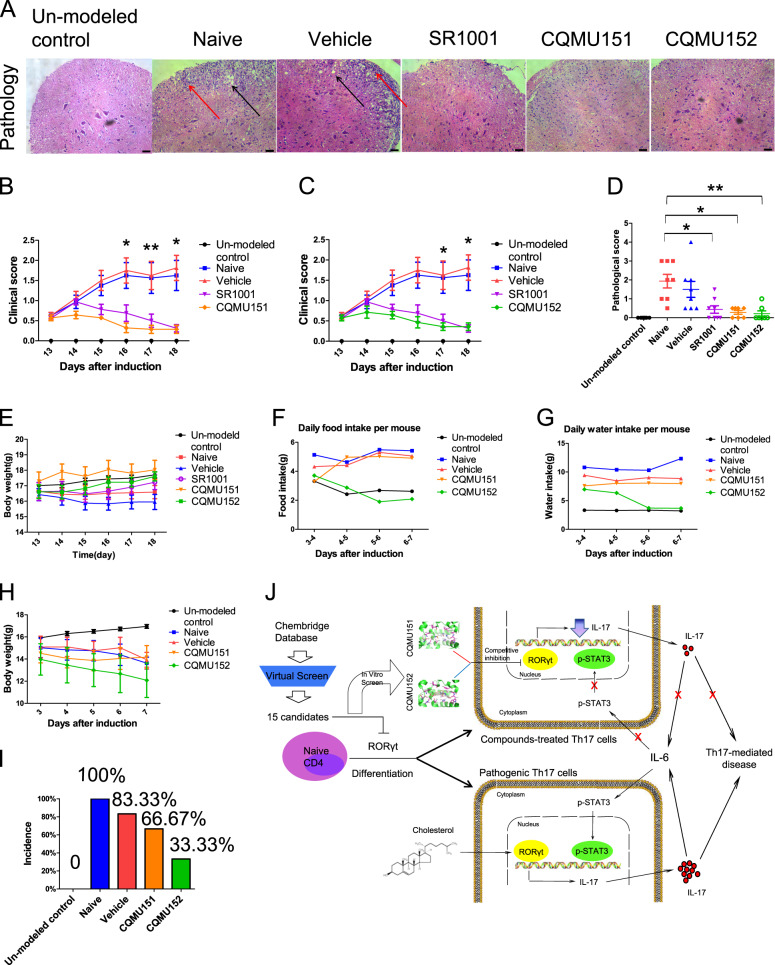
CQMU151 and CQMU152 can also inhibit the onset of EAE and T1DM. **a** Spinal cord section on day 18 of each group. Black arrows represent demyelinating changes, and red arrows represent inflammatory cell infiltration. Scale bar, 40 µm. **b**, **c** Clinical score of CQMU151 and CQMU152, respectively (*P < 0.05, **P < 0.01 versus the vehicle group with Kruskal–Wallis, un-modeled control, naive, vehicle and SR1001 group: *n* = 8, compounds groups: *n* = 7). **d** Pathological score of CQMU151 and CQMU152 (*P < 0.05, **P < 0.01 versus the control group with Kruskal–Wallis, un-modeled control, naive, vehicle and SR1001 group: *n* = 8, compounds groups: *n* = 7). **e** Weight change of mice in each group. The weight of the mice was monitored daily from day 13 to 18. **f**, **g** Changes in the average daily food intake and water consumption of type 1 diabetes mice in each group were monitored from the onset of the third day. *n* = 6. **h** Daily weight change trend of mice in each group. *n* = 6. **i** Statistical map of the incidence of each group after modeling. *n* = 6. **j** Schematic of the screening process and in vivo action of CQMU151 and CQMU152.

Our results showed that the two compounds had a therapeutic effect in EAU by inhibiting the differentiation of Th17 cells. We expanded our studies to other experimental models of autoimmune disease including EAE and type 1 diabetes, both of which are known to be Th17-mediated^31,32^. SR1001, a therapeutically effective RORγt inverse agonist in EAE, was included as a positive control. A daily clinical scoring was performed after starting the treatment with the two compounds. On the 18th day after immunization, the pathological scores were assessed. The results showed that the clinical scores and pathological scores of the treatment groups were significantly lower than those of the control groups and none of the compounds caused significant in vivo toxicity (Fig. 6a–e). After treatment the mice showed fewer symptoms of paralysis of the extremities and weakness of the tail. Pathological sections also showed less inflammatory cell infiltration and less demyelinating lesions. In the type 1 diabetes model, treatment caused a lower disease incidence of disease and clinical symptoms of the mice in the treatment groups, such as changes in water consumption and food intake, were lower than those in the control groups (Fig. 6f–i). Data of the experiments described above are summarized in a flowchart (Fig. 6j).

## Discussion

This study is the first to discover small-molecule compounds for the treatment of autoimmune disease by combining high-throughput virtual screening targeting RORγt and in vivo validation. From the 15 candidate compounds, two compounds, CQMU151 and CQMU152, were found to have obvious therapeutic effects as shown by a combination of in vitro screening and in vivo experimental models of autoimmune disease such as EAU, EAE, and type 1 diabetes. Our findings suggest that these two compounds may be used for the treatment of Th17-mediated disease.

The present study showed that compound CQMU151 and CQMU152, inhibit the differentiation of the Th17 lymphocyte subpopulation, but has no effect on the differentiation of Th1 and Treg cell lineages in vitro. Although these two compounds can inhibit RORα, the comparison of inhibition rates shows that the inhibitory effects on RORα are smaller than its effect on RORγt and RORγ. Further in vivo study showed that these two compounds significantly reduced the severity of EAU as shown by a reduction of both clinical and pathological scores. The clinical score continued to rise the next day after starting the treatment, but gradually reduced and remained stable by the third day. Consistent with the in vitro results, both compounds specifically inhibited the differentiation of Th17 and its cytokine IL-17 without affecting the Th1 and Treg cells lineages. Our study showed similar therapeutic effects on EAU as a previous study where the drug TMP778 was use to target RORγt^20^.

The production of Th17 cell cytokines is dependent on RORγt, STAT3 transduction pathway^33^, whereby the SOCS protein is a key physiological inhibitor of the STAT signaling pathway^34,35^. Compound CQMU151 and CQMU152 can inhibit the production of IL-17 by competitive binding with the endogenous ligand of RORγt such as steroids. The decreased expression of IL-17 found after treatment with the two compounds we identified subsequently may lead to a reduced expression of p-STAT3. SOCS3 inhibits STAT3 via the JAK-STAT pathway, but our results showed that the two compounds do not act on the SOCS-STAT pathway and that it is unlikely that they act directly on STAT3. Because our compounds affect the expression of IL-17, and the expression of IL-17 can further promote the expression of IL-17/IL-6/STAT3 loop^36,37^. We thus speculate that the decreased expression of p-STAT3 may be caused by the decrease of IL-17 blocking the positive feedback loop. This is a speculation and other mechanisms may also play a role. We cannot completely exclude the possibility that the compounds itself may inhibit phosphorylation of Y705 and further studies are needed to definitely prove our proposed mechanism of action.

Th17 cells are an important pathogenic factor for many autoimmune diseases, so we suspected that our RORγt inverse agonists could also affect other Th17 cell-mediated diseases. Our results showed that the clinical symptoms and histopathological lesions of EAE mice were significantly suppressed in our treatment groups. Compared with the existing compound SR1001, the two compounds can achieve the same or an even better therapeutic effect on EAE. Moreover, the therapeutic effects of these two compounds were also shown in type 1 diabetes. These two compounds could thus be useful in the treatment of a large variety of diseases that are caused by Th17 cells.

The BRB plays an important role in maintaining immune homeostasis in the retinal microenvironment, and the onset of EAU is usually accompanied by damage to the BRB, which is manifested by extensive leakage of blood vessels in the retina^38,39^. Our results showed that the treatment with these two compounds was associated with a maintained integrity of the BRB and that it reduced the pathological changes of retinal vascular leakage by upregulating the expression of tight junction proteins such as ZO-1. These effects are probably due to the fact that the treatment affected TH17 infiltration into the retina and that the observations on the BRB are a consequence of reduced inflammation. Taken together, it suggests that our compounds can significantly inhibit the infiltration of Th17 cells into the retina in EAU, thereby reducing local inflammation as evidenced by the fact that the expression of tight junction proteins and integrity of the BRB remain unaffected.

In this study, two compounds targeting RORγt were discovered by virtual screening and in vitro validation and were found to reduce the severity of multiple autoimmune diseases in vivo. In terms of mechanism, we found that these two compounds can inhibit the production of IL-17 by competitive binding with the endogenous ligand of RORγt and could then maintain the integrity of the BRB by reducing the infiltration of Th17 cells into the retina. In summary, the two novel compounds described in this study target RORγt, thereby inhibiting the pathogenic role of Th17 cells. Our findings may provide a new therapeutic strategy for Th17 cell-mediated diseases.

## Supplementary information

Supplementary figure legends

Supplementary figure 1

Supplementary figure 2

Supplementary figure 3

Supplementary figure 4

Supplementary tables
