# Supplementary material for: Small molecules targeting RORγt inhibit autoimmune disease by suppressing Th17 cell differentiation
Source: Cell Death Dis. 2020 Aug 22;11(8):697. doi: 10.1038/s41419-020-02891-2 (PMC7443190; doi:10.1038/s41419-020-02891-2)
Supplement: Supplementary file 1 — Supplementary figure legends [file 41419_2020_2891_MOESM1_ESM.docx]

**Supplementary Figure 1：The predicted binding modes.** (A-D) Compounds Z29584243, Z367373760, Z395316366, and Z56994861 bind in the RORγt protein’s ligand-binding domain. Compounds are shown as grays sticks. Key residues are shown as purple sticks. Hydrogen bonds interaction between compounds and protein are represented as yellow dashed line. π-π interactions are represented as cyan dashed line.

**Supplementary Figure 2: Effects of CQMU151 and CQMU152 on Th1 and Treg cells.** (A-C) The flow cytometry results of Th1 cell induction control group, CQMU151 and CQMU152 intervention group. (D-F) The flow cytometry results of Treg cell induction control group, CQMU151 and CQMU152 intervention group.

**Supplementary Figure 3: CQMU151 and CQMU152 treat disease rather than delay peak onset.** (A) Representative images of slit lamp photography of mice in each group on the 21st day after immunization. (B) Representative images of H&E staining of eyeball sections of mice in each group on the 21st day after immunization. (C-D) The clinical and pathological scores of each group on the 21st day after immunization

**Supplementary Figure 4: CQMU151 and CQMU152 cannot form a close dock with STAT3.** (A) Predicted binding mode of compounds CQMU151 in the RORγt ligand binding pocket. (B) Predicted binding mode of compounds CQMU151 in the STAT3 SH2 domain. (C-D) Predicted binding mode of CQMU151 and CQMU152 in the SH2 domain of STAT3, respectively. Compound CQMU151 is displayed in gray sticks. *cis-* and *trans-* isomer of compound CQMU152 are displayed as wheat and gray sticks respectively. Key residues are shown as purple sticks. Hydrogen bonds interaction between compounds and protein are represented as yellow dashed line.

**Supplementary Table 2**: Fifteen compounds intervene in the process of inducing Th17 cells with concentrations of 10 μM, 50 μM, and 100 μM, respectively. The first five are compounds that achieve 50% inhibition. Symbol(-) indicates no inhibition.
