# Supplementary figures and images for: Small molecules targeting RORγt inhibit autoimmune disease by suppressing Th17 cell differentiation
Source: Cell Death Dis. 2020 Aug 22;11(8):697. doi: 10.1038/s41419-020-02891-2 (PMC7443190; doi:10.1038/s41419-020-02891-2)

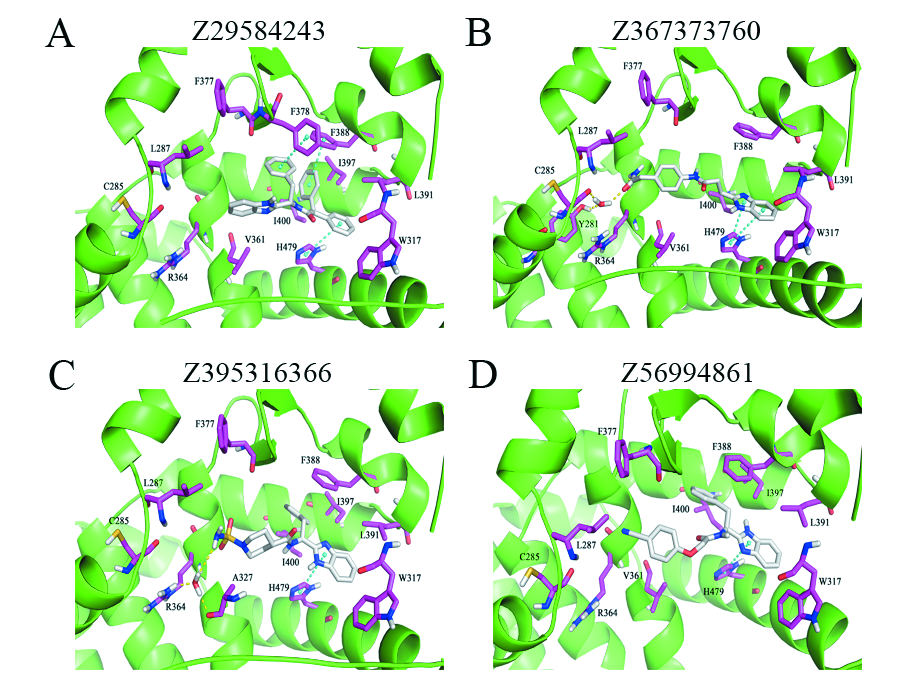

Supplement: Supplementary file 2 — Supplementary figure 1 [file 41419_2020_2891_MOESM2_ESM.tif]

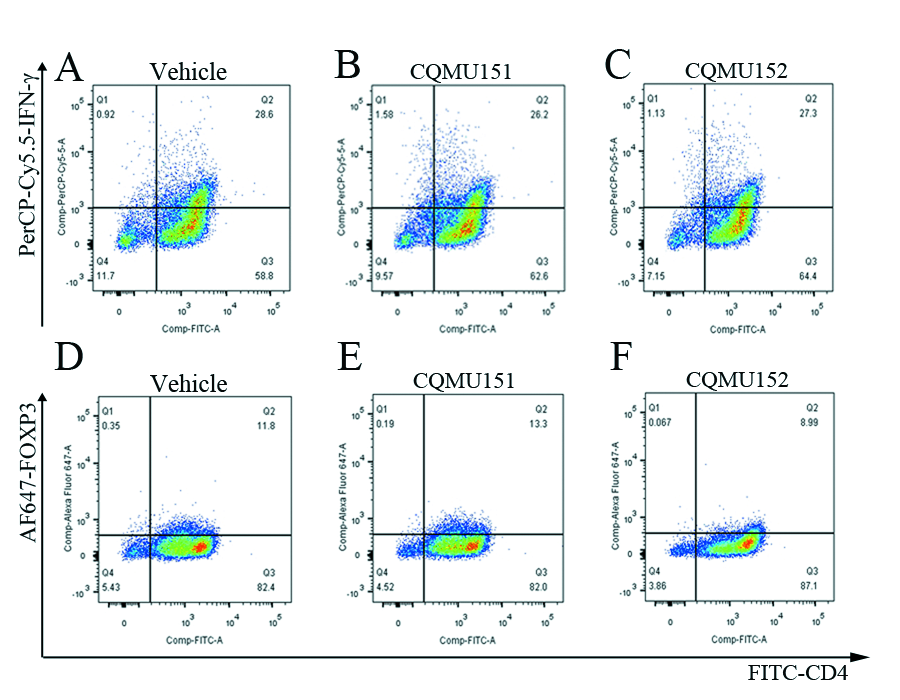

Supplement: Supplementary file 3 — Supplementary figure 2 [file 41419_2020_2891_MOESM3_ESM.tif]

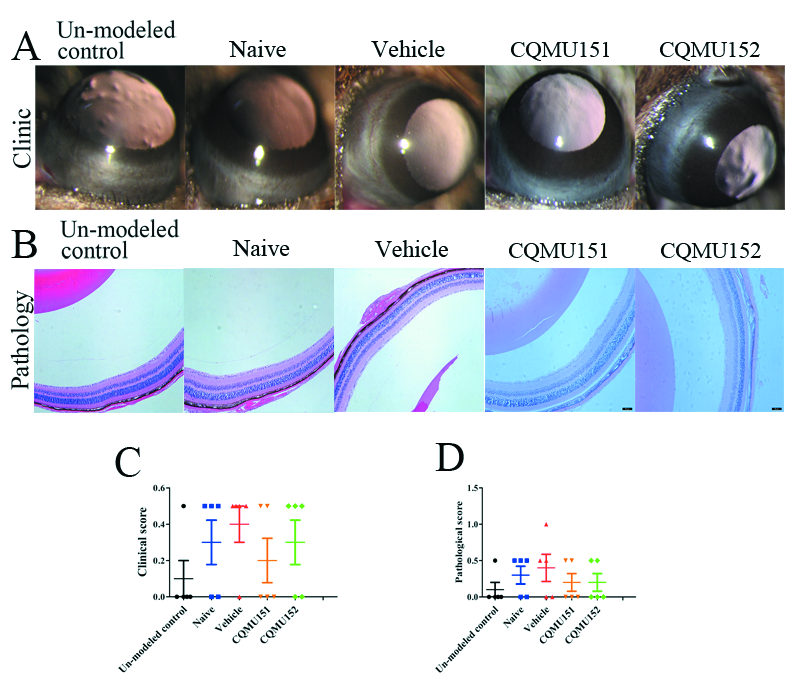

Supplement: Supplementary file 4 — Supplementary figure 3 [file 41419_2020_2891_MOESM4_ESM.tif]

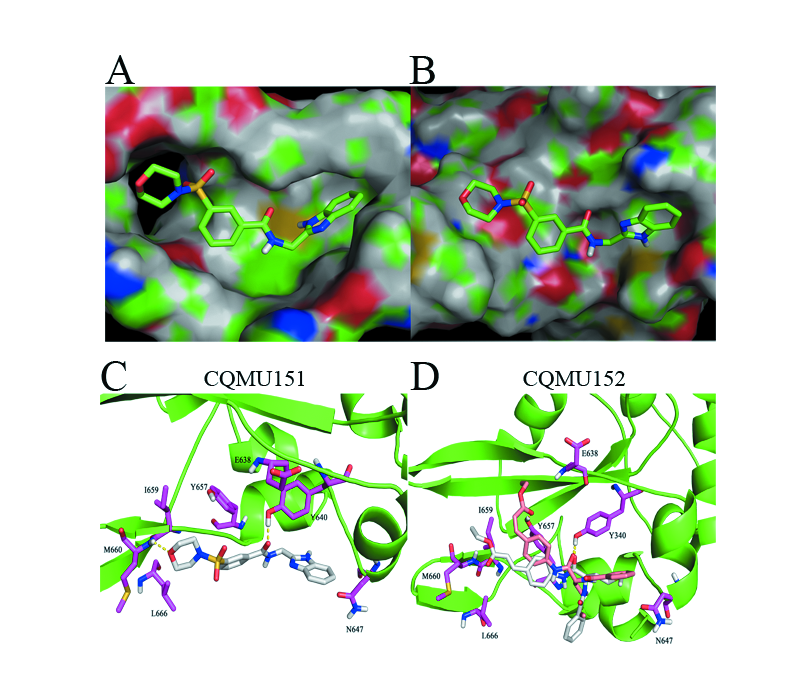

Supplement: Supplementary file 5 — Supplementary figure 4 [file 41419_2020_2891_MOESM5_ESM.tif]
