# Supplementary material for: Small molecules targeting RORγt inhibit autoimmune disease by suppressing Th17 cell differentiation
Source: Cell Death Dis. 2020 Aug 22;11(8):697. doi: 10.1038/s41419-020-02891-2 (PMC7443190; doi:10.1038/s41419-020-02891-2)
Supplement: Supplementary file 6 — Supplementary tables [file 41419_2020_2891_MOESM6_ESM.docx]

**Supplementary Table 1 Fifteen candidate compounds and their corresponding Glide Scores**

| Compound | Structure | XP  GScore | Compound | Structure | XP  GScore |
| --- | --- | --- | --- | --- | --- |
| Z26782034  （CQMU151） |  | -9.60 | Z167626774  （CQMU152） |  | -9.40(Z)  -9.49(E) |
| Z29584243 |  | -12.05 | Z109399880 |  | -13.00 |
| Z221558236 |  | -11.46 | Z367373760 |  | -9.89 |
| Z164787236 |  | -9.96 | Z395316366 |  | -11.71 |
| Z56994861 |  | -11.87 | Z29582801 |  | -12.61 |
| Z99372128 |  | -12.32 | Z27506774 |  | -12.27 |
| Z28832547 |  | -10.83 | Z317872448 |  | -9.12 |
| Z106765262 |  | -12.45 |  |  |  |

**Supplementary Table 2 The Proportion of Th17 cells of 15 compounds (%)**

| Compound | 10μM | 50μM | 100μM | Maximum inhibition rate(%) | Non-intervention control group |
| --- | --- | --- | --- | --- | --- |
| CQMU151 | 4.72 | 3.62 | 2.41 | 67.08 |  |
| CQMU152 | 5.04 | 0.29 | 2.48 | 96.04 |  |
| Z29584243 | 4.11 | 1.68 | 0.67 | 90.85 |  |
| Z164787236 | 8.77 | 5.82 | 2.11 | 71.17 |  |
| Z56994861 | 6.57 | 2.45 | 1.17 | 84.02 |  |
| Z109399880 | 7.96 | 6.58 | 6.24 | 14.75 |  |
| Z221558236 | 7.80 | 7.42 | 9.07 | - | 7.32 |
| Z367373760 | 6.33 | 10.5 | 4.61 | 37.02 |  |
| Z395316366 | 4.11 | 5.31 | 4.89 | 43.85 |  |
| Z29582801 | 8.35 | 7.77 | 7.21 | - |  |
| Z99372128 | 12.4 | 9.09 | 11.8 | - |  |
| Z27506774 | 11.6 | 10.7 | 10.2 | - |  |
| Z28832547 | 10.2 | 13.7 | 8.72 | - |  |
| Z317872448 | 12.4 | 8.64 | 13.2 | - |  |
| Z106765262 | 10.1 | 9.03 | 6.86 | 6.28 |  |

**Supplementary Table 3 Glide Scores of COMU151 and CQMU152 against RORγt and STAT3**

| Compound | GScore (RORγt) | GScore (STAT3) |
| --- | --- | --- |
| CQMU151 | -9.60 | -3.88 |
| CQMU152 | -9.40(Z), -9.49(E) | -3.56(Z), -2.01 |
